# Qualidade de dados antropométricos de crianças menores de 5 anos no Sistema de Vigilância Alimentar e Nutricional, 2008-2020 

**DOI:** 10.1590/0102-311XPT070523

**Published:** 2024-02-02

**Authors:** Iolanda Karla Santana dos Santos, Wolney Lisboa Conde

**Affiliations:** 1 Faculdade de Saúde Pública, Universidade de São Paulo, São Paulo, Brasil.; 2 Fundação Universidade Federal do ABC, Santo André, Brasil.

**Keywords:** Antropometria, Vigilância Alimentar e Nutricional, Estado Nutricional, Atenção Primária à Saúde, Confiabilidade dos Dados, Anthropometry, Food and Nutritional Surveillance, Nutritional Status, Primary Health Care, Data Accuracy, Antropometría, Vigilancia Alimentaria y Nutricional, Estado Nutricional, Atención Primaria de Salud, Exactitud de los Datos

## Abstract

O planejamento, o monitoramento e a avaliação das ações de alimentação e nutrição dependem de estimativas confiáveis realizadas a partir de dados antropométricos de qualidade adequada. O objetivo deste estudo foi analisar a qualidade de dados antropométricos de crianças menores de 5 anos no Sistema de Vigilância Alimentar e Nutricional (SISVAN) no período de 2008 a 2020. A amostra compreendeu 23.453.620 crianças menores de 5 anos. Inicialmente, avaliamos a distribuição de valores faltantes e de valores fora do espectro do equipamento e calculamos o índice de preferência de dígito para peso e altura. Os índices nutricionais altura para idade (A-I), peso para idade (P-I) e índice de massa corporal para idade (IMC-I) foram calculados com a utilização do padrão de crescimento da Organização Mundial da Saúde, de 2006. Em seguida, sinalizamos os valores biologicamente implausíveis (VBI) e calculamos o desvio padrão (DP) dos índices nutricionais. Para cada município, calculamos a média e o DP de A-I e P-I e plotamos os valores de DP em função da média. Em todas as Unidades Federativas, o índice de preferência de dígito alcançou valor mínimo de 80 para altura e 20 para peso. Para os três índices nutricionais, houve redução da frequência de VBI no período de 2008 a 2020. Mesmo após a exclusão dos VBI, identificamos elevada variabilidade para os três índices nutricionais. Os indicadores avaliados demonstraram baixa qualidade da mensuração principalmente nas regiões Norte e Nordeste. Nossos resultados indicam qualidade insuficiente dos dados antropométricos em crianças menores de 5 anos e reforçam a necessidade de investimento em ações para o aprimoramento da coleta e do registro das informações antropométricas.

## Abstract (en)

The planning, monitoring, and evaluation of food and nutrition actions depend on reliable estimates based on adequate anthropometric data. The study aimed to analyze the quality of anthropometric data of children aged under 5 years in the Brazilian National Food and Nutrition Surveillance System (SISVAN) from 2008 to 2020. The sample comprised 23,453,620 children aged under 5 years. Initially, we evaluated the distribution of missing values and values outside the spectrum of the instrument, and calculated the digit preference index for weight and height. The nutritional indexes height for age (HAZ), weight for age (WAZ), and body mass index for age (BAZ) were calculated according to the World Health Organization 2006 child growth standards. Then, we identified the biologically implausible values (BIV) and calculated the standard deviation (SD) of the nutritional indexes. For each municipality, we calculated the mean and SD of HAZ and WAZ; and plotted the SD values as a function of the mean. In all Federative Units, the digit preference index reached a minimum value of 80 for height and 20 for weight. For the three nutritional indexes, there was a reduction in the frequency of BIV in the 2008-2020 period. Even after the exclusion of BIV, we identified high variability for the three nutritional indexes. The indicators evaluated showed low quality of measurement, especially in the North and Northeast regions. Our results indicate insufficient quality of anthropometric data in children aged under 5 years, and reinforce the need to invest in actions to improve the collection and recording of anthropometric information.

## Abstract (es)

La planificación, monitoreo y evaluación de acciones de alimentación y nutrición dependen de estimaciones confiables realizadas a partir de datos antropométricos de calidad adecuada. El objetivo del estudio fue analizar la calidad de datos antropométricos de niños menores de 5 años en el Sistema de Vigilancia Alimentaria y Nutricional (SISVAN) entre los años 2008 y 2020. La muestra se compuso de 23.453.620 niños menores de 5 años. Al principio, evaluamos la distribución de valores faltantes y de valores fueras del espectro del equipo, y calculamos el índice de preferencia de dígito para peso y altura. Los índices nutricionales altura para edad (A-E), peso para edad (P-E) e índice de masa corporal para edad (IMC-E) se calcularon utilizando el patrón de crecimiento de la Organización Mundial de la Salud de 2006. Luego, indicamos los valores biológicamente inverosímiles (VBI) y calculamos la desviación estándar (DE) de los índices nutricionales. Para cada municipio, calculamos la media y la DE de A-E y P-E; y representamos los valores de DE en función de la media. En todas las Unidades Federativas, el índice de preferencia de dígito alcanzó el valor mínimo de 80 para altura y 20 para peso. Para los tres índices nutricionales, hubo una disminución de la frecuencia de VBI entre los años de 2008 y 2020. Incluso tras excluir los VBI, identificamos una alta variabilidad para los tres índices nutricionales. Los indicadores evaluados demostraron una baja calidad de medición, sobre todo en las regiones Norte y Nordeste. Nuestros resultados indican una calidad insuficiente de datos antropométricos en niños menores de 5 años y fortalecen la necesidad de inversión en acciones para mejorar la recolección y registro de las informaciones antropométricas.

## Introdução

O monitoramento do crescimento e do desenvolvimento infantil na atenção primária à saúde (APS) é preconizado pelo Ministério da Saúde ^1^. A vigilância alimentar e nutricional cumpre esse papel no Brasil e está inserida na Política Nacional de Alimentação e Nutrição (PNAN) como uma de suas diretrizes ^2^. A vigilância alimentar e nutricional corresponde às ações de monitoramento de tendências da alimentação e do estado nutricional e de seus fatores determinantes na população brasileira ^3^^,^^4^. Dessa maneira, as informações produzidas no âmbito da APS são fundamentais para formulação, implementação e monitoramento de políticas públicas de alimentação e nutrição ^5^. Nos acompanhamentos realizados pelos profissionais da saúde na população pediátrica é esperado que os dados antropométricos aferidos sejam classificados segundo o padrão de crescimento da Organização Mundial da Saúde (OMS) ^6^, permitindo a identificação precoce de agravos nutricionais para, assim, orientar o cuidado adequado à criança ^7^.

O Sistema Único de Saúde (SUS) adota o conceito ampliado de vigilância alimentar e nutricional, no qual são utilizadas fontes administrativas e de inquéritos populacionais ^4^. No Sistema de Vigilância Alimentar e Nutricional (SISVAN), principal fonte administrativa de vigilância alimentar e nutricional no Brasil, são registrados dados das pessoas atendidas na APS do SUS. Dados antropométricos e marcadores de consumo alimentar registrados no e-SUS APS e dados antropométricos registrados no sistema de gestão do Programa Bolsa Família na saúde são compilados no SISVAN ^7^. Os dados agregados do SISVAN estão disponíveis para os gestores e em relatórios públicos para a sociedade civil acompanhar as tendências da alimentação e do estado nutricional da população brasileira (https://sisaps.saude.gov.br/sisvan/).

A qualidade dos dados em sistemas de informação em saúde é um construto multidimensional e engloba, mas não se limita, as dimensões de acessibilidade, clareza metodológica, cobertura, completitude, confiabilidade, consistência, não duplicidade, oportunidade e validade ^8^. A confiabilidade dos dados se refere ao grau de concordância entre medidas diferentes em condições similares; a consistência diz respeito à coerência entre variáveis que têm algum tipo de relação; e a validade, ao grau em que o dado mensura o que pretende mensurar ^8^. De acordo com a OMS, a qualidade dos dados antropométricos deve ser analisada para verificar se há algum aspecto que possa incorrer em estimativas enviesadas ^9^. Os vieses podem ser aleatórios ou sistemáticos e, em geral, são decorrentes de imprecisão na aferição antropométrica ^9^.

Estudos com dados do SISVAN com o objetivo de avaliar a cobertura ou as tendências do estado nutricional em diferentes fases e eventos de vida foram publicados nos últimos anos ^10^^,^^11^^,^^12^^,^^13^^,^^14^^,^^15^. Pesquisas sobre a confiabilidade dos dados antropométricos são menos frequentes ^16^^,^^17^. Avaliar a qualidade dos dados antropométricos é relevante, na medida em que a elevada variabilidade ou sobredispersão pode impactar a estimativa dos indicadores do estado nutricional na população infantil, uma vez que os diagnósticos são realizados com base nos valores extremos das distribuições - e distribuições com excesso de variabilidade tendem a ter caudas mais longas e densas ^18^. A avaliação do crescimento infantil é complexa porque a variação intraindividual em altura e em peso atribuída ao crescimento normal necessita ser diferenciada da variação implausível que possivelmente é consequência de erro na mensuração ^19^.

A presença de erro de medida é frequentemente subvalorizada. Grellety & Golden ^18^ questionam duas premissas difundidas no meio científico: a primeira é a de que o aumento do tamanho da amostra automaticamente melhoraria a precisão das estimativas; e a segunda é a de que virtualmente os erros aleatórios são neutros, ou seja, valores sobrestimados anulariam os efeitos dos valores subestimados. Na antropometria, o erro de medida varia em função de quatro componentes: a pessoa (fase e/ou evento de vida e estrutura corporal), o instrumento de mensuração (adequado para a medida e calibrado), a padronização da técnica de mensuração e o desempenho profissional ^20^. Alguns indicadores têm sido utilizados para a avaliação da qualidade de dados antropométricos em inquéritos populacionais para determinar se os resultados podem ter algum tipo de viés, um impacto em termos de interpretação ou mesmo um limite em sua utilização ^9^. Os principais indicadores são completitude, razão sexo, arredondamento de idade, preferência de dígito, valores implausíveis e características da distribuição (média, desvio padrão - DP, assimetria e curtose) ^9^^,^^21^^,^^22^.

Considerando a importância da avaliação do estado nutricional para o monitoramento do crescimento, o impacto do erro de medida na prevalência dos indicadores nutricionais e a lacuna na produção científica em relação à magnitude e à distribuição dos indicadores da qualidade dos dados antropométricos na APS no Brasil, o objetivo deste estudo foi analisar a qualidade de dados antropométricos de crianças menores de 5 anos no SISVAN no período de 2008 a 2020.

## Métodos

**Figura 1:**
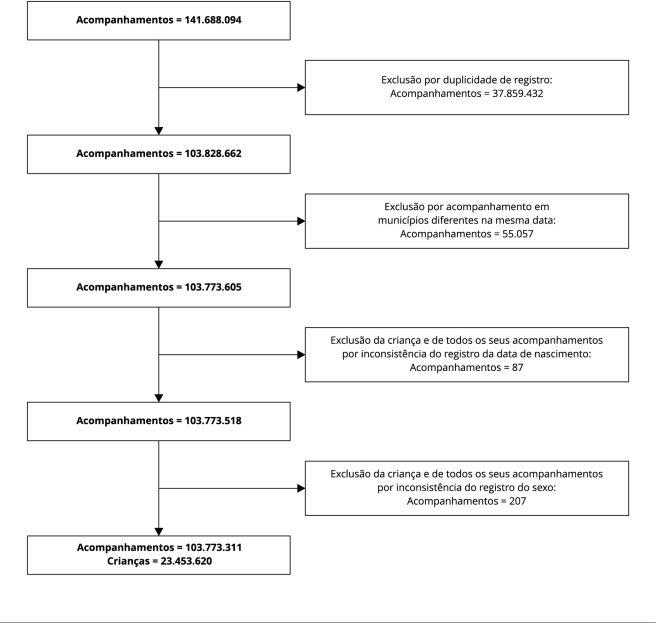
Fluxograma de seleção da amostra. Sistema de Vigilância Alimentar e Nutricional (SISVAN), Brasil, 2008-2020.

Este é um estudo transversal com dados do SISVAN do período de 2008 a 2020. As crianças menores de 5 anos foram selecionadas a partir da base de dados de acompanhamentos antropométricos de crianças e adolescentes disponibilizada pelo Ministério da Saúde. Na base de dados, havia 141.688.094 registros de crianças menores de 5 anos. Os registros duplicados foram excluídos, considerando as variáveis município, ano de referência, código de identificação da criança e data do acompanhamento. Em seguida, registros antropométricos coletados na mesma data para a mesma criança em municípios diferentes foram deletados. Algumas inconsistências foram identificadas. Para a mesma criança foram observados diferentes preenchimentos nos campos “data de nascimento” e “sexo” em verificação longitudinal. Nesses casos, decidimos excluir todos os registros da criança. Ao fim das etapas de seleção da amostra descritas na Figura 1, a população de estudo compreendeu 23.453.620 crianças menores de 5 anos, das quais foi selecionado o primeiro acompanhamento. Neste estudo, foi selecionado o primeiro registro de cada criança para minimizar o efeito dos acompanhamentos anteriores sobre a qualidade dos dados antropométricos.

Para a caracterização dos indicadores de qualidade da mensuração segundo região, Unidade Federativa (UF), faixa etária e ano de referência, adaptamos as recomendações da OMS estabelecidas para inquéritos ^9^:

(i) Frequência de valores faltantes para as variáveis peso e altura, além da própria ausência do valor, recodificamos o valor 0 como valor faltante;

(ii) Valores de altura acima de 200cm e peso acima de 200kg foram considerados como valores fora do espectro do equipamento. Esses valores foram determinados com base nas especificações de equipamentos contidas no *Manual Orientador para Aquisição de Equipamentos Antropométricos* da Secretaria de Atenção à Saúde do Ministério da Saúde ^23^. Os valores fora do espectro do equipamento foram excluídos antes do cálculo do índice para avaliação da preferência de dígito;

(iii) Preferência de dígito é a distribuição enviesada do último dígito. O índice de dissimilaridade, medida operacional da preferência de dígito, foi estimado pela fórmula: $\sum_{i=1}^{10}|{frequênciaobservada}_{i}-{frequênciaesperada}_{i}|/2$ . Consideramos como dígito terminal para altura em centímetros o número após a vírgula (p.ex.: se a altura aferida de uma criança foi 100,8cm, o dígito terminal é igual a 8). Para dígito terminal para peso em quilogramas, também consideramos o número após a vírgula (portanto, se o peso da criança foi 9,2kg, o dígito terminal é igual a 2 ^24^). Em distribuições não enviesadas de peso ou altura, é esperado que cada algarismo decimal tenha frequência de 10%. O índice de dissimilaridade varia de 0 a 90 e representa o percentual de observações que precisam ser redistribuídas entre os dígitos terminais para alcançar uma distribuição uniforme. Em uma amostra na qual as medidas foram coletadas de maneira apropriada, o recomendado é que esse índice não seja superior a 20 ^9^;

(iv) Valores biologicamente implausíveis (VBI) são valores que estão fora de um intervalo especificado e que são incompatíveis com a vida. Para o cálculo do indicador de frequência de VBI, inicialmente calculamos os índices nutricionais altura para idade (A-I), peso para idade (P-I) e índice de massa corporal para idade (IMC-I) para cada criança, com a utilização do padrão de crescimento da OMS ^6^. Para a sinalização dos VBI, os pontos de corte em DP em relação à mediana da população de referência foram < -6 DP ou > +6 DP para A-I, < -6 DP ou > +5 DP para P-I e < -5 DP ou > +5 DP para IMC-I. Antes do cálculo do IMC, foram excluídos os valores de peso e altura identificados como VBI de acordo com a sinalização nos índices P-I e A-I. Para a OMS, em uma amostra com qualidade de mensuração adequada, a frequência de VBI deve ser inferior a 1% ^25^;

(v) O DP dos índices nutricionais analisado neste estudo foi calculado após a exclusão dos VBI. A variabilidade aceitável para os dados antropométricos foi apresentada pela OMS no seu documento técnico de uso e interpretação da antropometria ^25^. A OMS estimou esses valores a partir de bases de dados nem sempre correspondentes a amostras representativas de suas respectivas populações. Adicionalmente, a referência do crescimento aplicada a essas amostras foi a do Centro Nacional de Estatísticas de Saúde dos Estados Unidos (NCHS)/OMS de 1978. O uso da referência NCHS/OMS de 1978 produziu espectro de validade mais estreito para as medidas antropométricas, levando à exclusão de um número superior de observações. Essas exclusões seriam diferentes caso o padrão de crescimento da OMS tivesse sido aplicado nas estimativas. A partir desse conjunto de informações, e dados os limites observados, foram propostas novas faixas de adequação para o DP calculado a partir de dados de altura e peso de crianças menores de 5 anos de 158 pesquisas de demografia e saúde (*Demographic and Health Surveys* - DHS) do período de 1986 a 2018 ^26^, harmonizados segundo o padrão de crescimento da OMS ^6^. Após a exclusão dos VBI, calculamos a média e o DP dos índices nutricionais A-I, P-I e IMC-I para cada inquérito. Em nossa base agregada de inquéritos, utilizamos regressão quantílica simultânea com os percentis 5, 50 e 95. Nessa regressão, o escore do DP (Z) foi a variável dependente e a média, a variável independente. Com base nos parâmetros da regressão, estimamos os valores do DP para as médias -2 Z e +1 Z. Os valores do desvio-padrão estimados foram: 1,1 a 1,4 para A-I, 1,0 a 1,2 para P-I e 1,0 a 1,1 para IMC-I, respectivamente.

Em seguida, para cada município do Brasil, calculamos a média e o DP dos índices nutricionais A-I e P-I. Esses valores de DP foram plotados em função da média. Antes disso, excluímos municípios com menos de 100 crianças (n = 13), média < -2 Z ou > +1 Z (n = 3) e DP < 0,5 ou > 2,5 (n = 11), totalizando 5.543 municípios. Cada círculo representa um município, e o diâmetro do círculo é proporcional ao número de observações. As análises foram realizadas no programa Stata, versão 17.0 (https://www.stata.com).

Este estudo foi apreciado e aprovado pelo Comitê de Ética e Pesquisa da Faculdade de Saúde Pública, Universidade de São Paulo (CAAE 53220716.8.0000.5421 e parecer nº 4.607.143, de 23 de março de 2021).

## Resultados

A amostra compreendeu 50,4% de crianças do sexo feminino e 49,6% do sexo masculino. No primeiro acompanhamento, 53,1% das crianças tinham até 2 anos de idade. Na Tabela 1, apresentamos os indicadores da qualidade da mensuração segundo região geográfica e UF. A frequência de valores faltantes foi baixa, sendo inferior a 0,5% para altura e peso, assim como a frequência de valores fora do espectro do equipamento. O índice de preferência de dígito foi maior do que 80 para altura em todas as UFs. A preferência de dígito foi menos acentuada para peso, mas ainda assim superior a 20, e o menor valor foi observado no Distrito Federal (20,91). A frequência de VBI foi superior a 1% em todas as regiões brasileiras para os índices nutricionais A-I e P-I. No Brasil, a frequência de VBI para A-I (4,14%) foi superior à de P-I (2,07%). Para algumas UFs (Distrito Federal, São Paulo, Paraná e Santa Catarina), a frequência de VBI para IMC-I foi inferior a 1%, mas é necessário destacar que, antes do cálculo dessa frequência, foram excluídos os valores de peso e altura sinalizados como biologicamente implausíveis de acordo com a sinalização nos índices P-I e A-I e, portanto, o IMC para algumas crianças não foi calculado. Os indicadores da qualidade da mensuração com valores mais próximos da faixa de adequação foram observados no Distrito Federal.

**Tabela 1:** Indicadores da qualidade dos dados antropométricos segundo região geográfica e Unidade Federativa (UF). Sistema de Vigilância Alimentar e Nutricional (SISVAN), Brasil, 2008-2020.

Região/UF	Valores faltantes		Valores fora do espectro		Preferência de dígito		VBI			DP		
	Altura	Peso	Altura	Peso	Altura	Peso	A-I	P-I	IMC-I	A-I	P-I	IMC-I
	%	%	%	%			%	%	%			
Centro-oeste	0,12	0,11	0,09	0,01	87,60	34,79	3,93	2,14	1,62	1,70	1,30	1,50
Distrito Federal	0,07	0,06	0,13	0,01	82,11	20,91	2,02	1,44	0,82	1,43	1,22	1,35
Goiás	0,14	0,14	0,07	0,02	88,20	40,32	4,77	2,45	2,10	1,79	1,34	1,57
Mato Grosso	0,18	0,13	0,09	0,01	87,98	35,62	4,27	2,31	1,62	1,70	1,30	1,50
Mato Grosso do Sul	0,06	0,05	0,13	0,01	88,34	30,56	2,98	1,77	1,18	1,66	1,27	1,45
Nordeste	0,15	0,13	0,06	0,02	88,27	47,70	5,14	2,25	2,27	1,85	1,37	1,65
Alagoas	0,14	0,12	0,03	0,01	88,60	49,47	5,03	2,25	2,24	1,84	1,39	1,65
Bahia	0,16	0,14	0,07	0,02	88,47	43,21	4,55	2,18	1,94	1,78	1,33	1,61
Ceará	0,15	0,14	0,02	0,01	88,33	47,98	4,97	2,01	2,30	1,82	1,35	1,64
Maranhão	0,18	0,16	0,06	0,01	88,81	64,44	6,38	2,33	2,77	1,96	1,41	1,72
Paraíba	0,12	0,11	0,05	0,06	88,03	39,81	4,30	2,40	1,71	1,75	1,33	1,56
Pernambuco	0,14	0,12	0,03	0,02	87,54	44,76	5,44	2,52	2,61	1,91	1,42	1,67
Piauí	0,13	0,11	0,02	0,01	88,27	48,77	5,16	2,13	1,98	1,78	1,33	1,61
Rio Grande do Norte	0,15	0,14	0,21	0,02	87,41	43,02	4,82	2,13	2,24	1,79	1,35	1,62
Sergipe	0,15	0,15	0,40	0,01	88,60	48,59	5,79	2,28	2,77	1,96	1,36	1,74
Norte	0,11	0,11	0,01	0,01	88,85	43,59	4,04	1,61	1,79	1,77	1,33	1,56
Acre	0,20	0,19	0,02	0,01	88,82	48,75	4,62	1,95	2,03	1,80	1,32	1,59
Amapá	0,11	0,11	0,00	0,01	88,61	30,86	3,68	1,45	1,62	1,68	1,27	1,49
Amazonas	0,15	0,14	0,01	0,00	89,15	38,64	4,00	1,63	1,81	1,76	1,36	1,55
Pará	0,09	0,09	0,01	0,01	88,76	48,25	4,01	1,46	1,82	1,78	1,33	1,58
Rondônia	0,06	0,08	0,01	0,00	88,44	43,08	3,48	1,47	1,58	1,68	1,26	1,53
Roraima	0,16	0,15	0,01	0,00	89,09	31,11	3,02	1,31	1,35	1,61	1,24	1,45
Tocantins	0,12	0,12	0,04	0,01	88,81	39,76	4,75	2,31	1,86	1,78	1,30	1,58
Sudeste	0,14	0,13	0,10	0,04	87,15	28,36	3,56	2,18	1,33	1,65	1,33	1,47
Espírito Santo	0,13	0,08	0,13	0,06	87,75	25,90	3,09	1,70	1,16	1,59	1,27	1,46
Minas Gerais	0,07	0,07	0,13	0,01	88,05	28,89	4,25	2,61	1,47	1,73	1,37	1,51
Rio de Janeiro	0,48	0,45	0,03	0,01	87,05	29,02	4,02	2,16	1,88	1,73	1,37	1,53
São Paulo	0,07	0,08	0,11	0,07	86,12	27,86	2,67	1,81	0,98	1,52	1,29	1,39
Sul	0,08	0,05	0,10	0,03	85,89	27,37	2,74	1,71	0,96	1,57	1,30	1,41
Paraná	0,08	0,06	0,07	0,02	86,26	25,62	2,93	1,89	0,94	1,57	1,28	1,40
Rio Grande do Sul	0,09	0,05	0,11	0,03	85,80	29,57	2,38	1,35	1,02	1,56	1,30	1,43
Santa Catarina	0,09	0,05	0,13	0,05	85,32	27,82	2,86	1,83	0,94	1,58	1,31	1,40
Brasil	0,13	0,12	0,08	0,02	87,65	37,86	4,14	2,07	1,71	1,74	1,34	1,55

A-I: altura para idade; DP: desvio padrão; IMC-I: índice de massa corporal para idade; P-I: peso para idade; VBI: valores biologicamente implausíveis.

A frequência de valores faltantes foi maior entre crianças menores de 2 anos, e o ano de 2016 apresentou distribuição de frequência para esse indicador fora do padrão observado para os demais anos. Para altura, a frequência de valores fora do espectro do equipamento diminuiu de maneira expressiva a partir de 2013, alcançando virtualmente 0 no último ano. O dígito preferencial no caso da altura foi 0. No caso do peso, houve preferência pelos dígitos 0 e 5. A preferência de dígito para altura se manteve superior a 80 durante todo o período. A preferência de dígito para peso seguiu uma gradação positiva em relação à idade, ou seja, quanto maior a faixa etária, maior o valor do índice. A preferência de dígito para peso diminuiu de 50,4 para 18,9 entre 2008 e 2020.

A frequência de VBI para A-I foi maior entre crianças menores de 2 anos. Na maioria dos anos de referência, a frequência de VBI para A-I foi maior na faixa etária de 12 a 23 meses. Para o índice P-I, observamos gradiente inverso em relação à idade, ou seja, quanto maior a faixa etária, menor a frequência de VBI. Na distribuição do IMC-I, os valores de VBI apresentaram formato de U invertido em relação à faixa etária. Para os três índices nutricionais, houve redução da frequência de VBI no período de 2008 a 2020: A-I passou de 4,5% para 2,5%; P-I, de 2,2% para 0,7%; e IMC-I, de 1,9% para 1% (Tabela 2).

**Tabela 2:** Indicadores da qualidade dos dados antropométricos segundo ano de referência e faixa etária. Sistema de Vigilância Alimentar e Nutricional (SISVAN), Brasil, 2008-2020.

Indicador/Faixa etária (meses)	2008	2009	2010	2011	2012	2013	2014	2015	2016	2017	2018	2019	2020
Valores faltantes - altura (%)													
0-11	0,22	0,14	0,07	0,10	0,11	0,00	0,00	0,01	0,60	0,00	0,00	0,03	0,01
12-23	0,09	0,06	0,03	0,07	0,05	0,00	0,00	0,01	1,96	0,00	0,00	0,11	0,12
24-35	0,04	0,06	0,02	0,05	0,03	0,00	0,00	0,01	0,68	0,00	0,00	0,16	0,22
36-47	0,02	0,04	0,02	0,05	0,02	0,00	0,00	0,01	1,26	0,00	0,00	0,21	0,23
48-59	0,01	0,03	0,02	0,04	0,01	0,00	0,00	0,01	1,16	0,00	0,00	0,19	0,23
Total	0,04	0,06	0,03	0,06	0,05	0,00	0,00	0,01	1,08	0,00	0,00	0,11	0,07
Valores faltantes - peso (%)													
0-11	0,04	0,02	0,01	0,03	0,02	0,00	0,00	0,05	0,88	0,01	0,00	0,03	0,01
12-23	0,01	0,01	0,00	0,01	0,01	0,00	0,00	0,02	1,72	0,00	0,00	0,09	0,09
24-35	0,00	0,01	0,00	0,01	0,00	0,00	0,00	0,01	0,87	0,00	0,00	0,15	0,18
36-47	0,00	0,01	0,01	0,01	0,00	0,00	0,00	0,01	0,91	0,01	0,00	0,19	0,20
48-59	0,00	0,01	0,00	0,01	0,00	0,00	0,00	0,02	0,86	0,00	0,00	0,17	0,20
Total	0,01	0,01	0,00	0,01	0,01	0,00	0,00	0,03	1,08	0,00	0,00	0,10	0,06
Valores fora do espectro - altura (%)													
0-11	0,68	0,53	0,52	0,49	0,41	0,02	0,00	0,00	0,00	0,00	0,00	0,01	0,00
12-23	0,20	0,24	0,19	0,24	0,15	0,01	0,01	0,00	0,00	0,00	0,00	0,01	0,01
24-35	0,13	0,19	0,15	0,16	0,12	0,01	0,01	0,00	0,00	0,00	0,00	0,01	0,00
36-47	0,12	0,24	0,17	0,21	0,15	0,01	0,01	0,00	0,00	0,00	0,00	0,01	0,00
48-59	0,08	0,20	0,16	0,17	0,17	0,01	0,01	0,00	0,00	0,00	0,00	0,00	0,00
Total	0,16	0,27	0,23	0,27	0,22	0,01	0,01	0,00	0,00	0,00	0,00	0,01	0,00
Valores fora do espectro - peso (%)													
0-11	0,79	0,04	0,03	0,01	0,03	0,01	0,00	0,00	0,00	0,00	0,00	0,01	0,00
12-23	0,21	0,02	0,02	0,00	0,02	0,00	0,00	0,00	0,00	0,00	0,00	0,01	0,00
24-35	0,13	0,01	0,01	0,01	0,00	0,00	0,00	0,00	0,00	0,00	0,00	0,00	0,00
36-47	0,15	0,02	0,01	0,01	0,00	0,01	0,00	0,01	0,00	0,00	0,00	0,00	0,00
48-59	0,14	0,03	0,02	0,02	0,01	0,02	0,02	0,01	0,01	0,00	0,00	0,00	0,00
Total	0,19	0,02	0,02	0,01	0,01	0,01	0,00	0,00	0,00	0,00	0,00	0,01	0,00
Preferência de dígito - altura													
0-11	85,07	84,07	84,98	83,74	83,41	89,60	89,96	87,99	86,65	88,30	82,50	86,11	81,38
12-23	89,09	88,63	89,15	88,76	88,41	89,88	89,98	89,22	89,20	89,54	79,11	88,16	85,82
24-35	89,50	88,99	89,40	89,09	88,89	89,90	89,92	89,57	89,45	89,67	78,95	88,56	87,15
36-47	89,69	89,23	89,57	89,27	89,18	89,91	89,93	89,70	89,58	89,74	78,25	88,73	87,96
48-59	89,77	89,39	89,60	89,29	89,18	89,94	89,92	89,74	89,59	89,74	78,39	88,83	88,21
Total	89,28	88,18	88,67	87,69	87,43	89,84	89,95	88,96	88,37	89,19	80,16	87,58	83,47
Preferência de dígito - peso													
0-11	23,22	20,12	23,25	19,58	17,09	24,98	25,57	22,04	19,53	22,66	18,57	20,44	10,13
12-23	44,82	41,79	44,70	42,75	40,78	44,61	42,98	40,98	39,47	39,01	31,07	35,77	28,12
24-35	51,97	49,07	51,49	49,28	47,84	49,75	47,94	48,16	45,72	44,11	35,76	40,34	35,70
36-47	53,87	51,53	52,87	50,67	49,24	50,96	47,91	51,27	48,44	45,53	36,48	42,03	37,85
48-59	54,87	52,73	53,80	51,44	49,59	51,41	48,42	54,54	51,68	46,94	37,55	43,39	39,03
Total	50,36	43,61	45,83	40,55	38,57	43,91	40,81	38,11	35,26	35,92	28,42	32,14	18,90
VBI A-I (%)													
0-11	7,93	6,96	7,29	6,14	5,68	6,75	6,78	4,83	3,48	4,58	3,42	3,08	2,39
12-23	7,19	7,39	7,41	7,05	6,49	6,79	6,89	5,37	4,15	3,98	3,58	4,66	3,60
24-35	5,00	5,02	4,99	4,99	4,55	4,68	4,88	3,80	3,44	2,61	2,30	3,06	2,77
36-47	3,48	3,62	3,36	3,41	3,16	3,34	3,54	2,65	1,42	1,53	1,39	1,75	1,24
48-59	2,77	3,00	2,81	2,74	2,57	2,61	2,80	1,90	0,83	0,95	0,93	1,24	0,93
Total	4,45	5,20	5,21	5,20	4,77	5,09	5,47	4,20	3,12	3,33	2,79	3,16	2,53
VBI P-I (%)													
0-11	5,35	4,62	4,66	3,99	4,13	6,65	5,93	3,70	1,93	1,73	0,92	1,05	0,55
12-23	3,30	3,20	3,24	3,16	2,97	3,55	3,56	2,84	1,26	0,88	0,83	1,43	0,91
24-35	2,13	2,08	2,00	2,05	2,01	2,17	2,38	1,90	1,13	1,00	0,85	1,03	0,89
36-47	1,71	1,78	1,66	1,73	1,57	1,94	2,09	1,74	0,79	0,67	0,69	0,93	0,65
48-59	1,40	1,52	1,39	1,47	1,27	1,60	1,77	1,36	0,51	0,38	0,52	0,70	0,48
Total	2,19	2,60	2,56	2,69	2,61	3,30	3,51	2,69	1,36	1,11	0,82	1,10	0,65
VBI IMC-I (%)													
0-11	1,20	1,09	1,23	1,04	0,98	1,23	1,33	1,09	0,86	1,22	1,13	1,31	0,60
12-23	2,10	2,04	2,14	1,99	1,85	2,07	2,12	1,86	1,86	2,03	1,87	2,08	1,50
24-35	2,22	2,22	2,39	2,20	2,09	2,24	2,28	2,08	2,14	2,29	2,13	2,31	1,98
36-47	2,04	2,16	2,12	2,09	1,94	2,05	2,11	1,91	1,87	2,06	2,02	2,30	2,14
48-59	1,65	1,83	1,74	1,67	1,55	1,70	1,74	1,51	1,56	1,74	1,58	1,80	1,79
Total	1,91	1,89	1,96	1,76	1,64	1,88	1,90	1,59	1,50	1,78	1,63	1,82	1,04
DP A-I													
0-11	1,84	1,80	1,84	1,79	1,75	1,81	1,82	1,74	1,67	1,77	1,73	1,79	1,53
12-23	1,95	1,96	1,98	1,94	1,88	1,92	1,93	1,86	1,87	1,88	1,85	1,90	1,74
24-35	1,80	1,81	1,84	1,81	1,76	1,77	1,76	1,72	1,72	1,74	1,71	1,76	1,67
36-47	1,67	1,69	1,69	1,69	1,63	1,65	1,64	1,59	1,58	1,61	1,59	1,62	1,56
48-59	1,53	1,56	1,55	1,53	1,50	1,53	1,52	1,45	1,44	1,49	1,47	1,49	1,47
Total	1,71	1,77	1,79	1,78	1,73	1,77	1,78	1,72	1,70	1,76	1,73	1,78	1,59
DP P-I													
0-11	1,39	1,40	1,40	1,41	1,41	1,41	1,41	1,35	1,34	1,37	1,35	1,38	1,30
12-23	1,31	1,33	1,33	1,32	1,29	1,33	1,33	1,31	1,34	1,32	1,32	1,35	1,31
24-35	1,32	1,33	1,35	1,35	1,32	1,35	1,35	1,33	1,33	1,35	1,33	1,34	1,34
36-47	1,30	1,33	1,33	1,34	1,32	1,35	1,35	1,33	1,33	1,34	1,32	1,33	1,36
48-59	1,27	1,32	1,33	1,33	1,31	1,34	1,34	1,33	1,33	1,33	1,33	1,33	1,38
Total	1,31	1,34	1,35	1,36	1,35	1,36	1,36	1,34	1,34	1,35	1,34	1,36	1,32
DP IMC-I													
0-11	1,52	1,51	1,53	1,50	1,48	1,54	1,56	1,52	1,46	1,54	1,52	1,54	1,39
12-23	1,58	1,58	1,60	1,57	1,54	1,59	1,59	1,55	1,55	1,57	1,55	1,56	1,48
24-35	1,60	1,61	1,64	1,61	1,59	1,62	1,61	1,58	1,57	1,59	1,56	1,57	1,54
36-47	1,54	1,57	1,58	1,57	1,54	1,57	1,55	1,54	1,52	1,54	1,52	1,51	1,53
48-59	1,47	1,50	1,51	1,50	1,48	1,51	1,49	1,48	1,47	1,49	1,48	1,47	1,51
Total	1,55	1,56	1,59	1,56	1,54	1,58	1,58	1,55	1,52	1,56	1,54	1,55	1,45

A-I: altura para idade; DP: desvio padrão; IMC-I: índice de massa corporal para idade; P-I: peso para idade; VBI: valores biologicamente implausíveis.

A sobredispersão para os três índices nutricionais permaneceu mesmo após a exclusão dos VBI. A dispersão dos dados foi menor entre as crianças de 36 a 59 meses para o índice A-I. Para o índice P-I, a dispersão seguiu um padrão uniforme em relação à faixa etária. Para o índice IMC-I, seguiu o padrão de U invertido, assim como observado na distribuição do VBI. Apesar de haver redução da frequência de VBI no período, a dispersão dos dados válidos se manteve estável, exceto para o ano de 2020 (Tabela 2).

**Figura 2:**
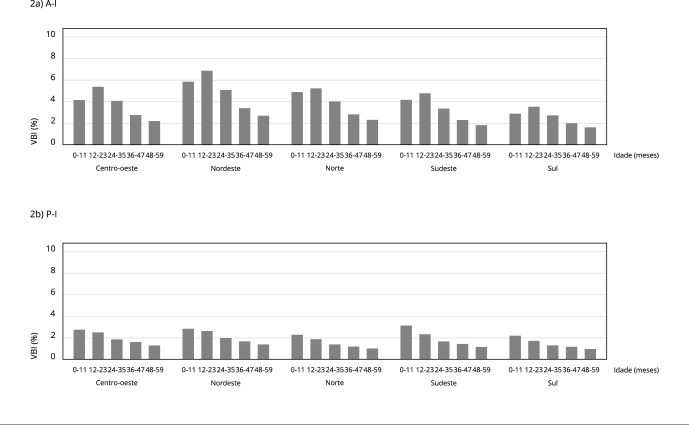
Frequência de valores biologicamente implausíveis (VBI) em altura para idade (A-I) e peso para idade (P-I) segundo faixa etária e região geográfica. Sistema de Vigilância Alimentar e Nutricional (SISVAN), Brasil, 2008-2020.

Na Figura 2, apresentamos a frequência de VBI para A-I e P-I segundo faixa etária e região geográfica. A Região Nordeste apresentou as maiores frequências de VBI para o índice A-I, enquanto as regiões Sul e Sudeste exibiram as menores frequências de VBI para esse índice. As diferenças percentuais entre as regiões geográficas são mais expressivas para a distribuição de A-I do que para a distribuição de P-I.

**Figura 3:**
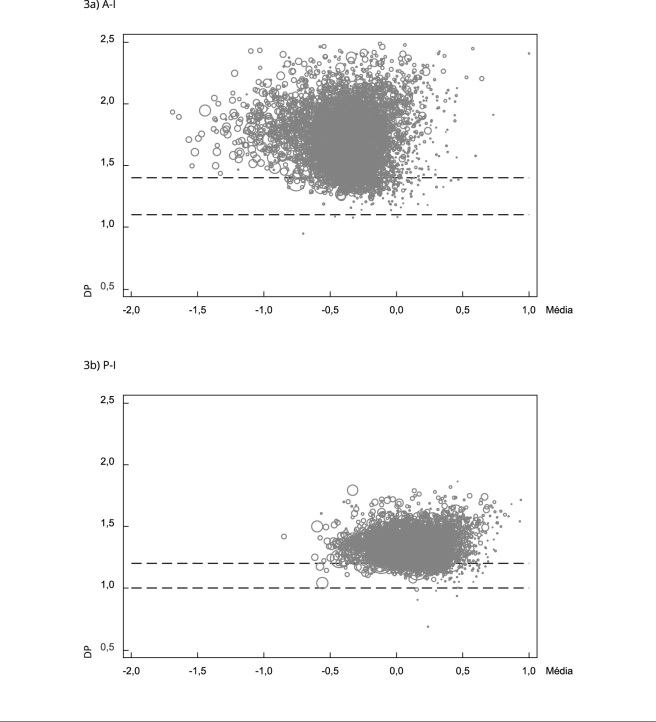
Desvio padrão (DP) em função da média para altura para idade (A-I) e peso para idade (P-I) de 5.543 municípios brasileiros. Sistema de Vigilância Alimentar e Nutricional (SISVAN), 2008-2020.

Na Figura 3, plotamos os valores de média e DP dos índices nutricionais A-I e P-I em cada município brasileiro; as linhas tracejadas representam a faixa de adequação para o DP estimada a partir dos dados da DHS. De modo geral, observamos que para A-I os municípios estão mais à esquerda da média do padrão de crescimento, enquanto para P-I os municípios estão em torno da média. A dispersão é mais acentuada para A-I do que para P-I. Em A-I, alguns municípios chegam a ter um valor igual ou superior a 2 DP, mesmo com uma média similar à do padrão de crescimento (igual a 0).

## Discussão

Os resultados indicam que: (1) a preferência de dígito foi maior do que 80 para altura e maior do que 20 para peso; (2) a maioria das mensurações da altura terminava com o algarismo 0; (3) a preferência de dígito para peso apresentou gradação positiva em relação à idade; (4) a frequência de VBI foi maior para altura do que para peso, e ambas foram superiores a 1%; (5) a distribuição dos valores de VBI para P-I foi inversa em relação à idade; (4) no período de 2008 a 2020, observamos redução da preferência de dígito para peso e dos VBI para os três índices nutricionais; (5) mesmo após a exclusão dos VBI, a sobredispersão foi nítida para os três índices nutricionais, em especial para A-I; e (6) as desigualdades regionais nas distribuições dos VBI foram menores nas regiões Sudeste e Sul.

As principais fontes de erro de medida são ausência de protocolo para execução da medida, aferição não padronizada, equipamento defeituoso ou descalibrado, equipamento inapropriado para a realização da técnica de mensuração e erro no registro ou na digitação dos dados ^18^^,^^19^^,^^27^. Erros de registro podem ser decorrentes de acréscimo ou troca de número, erro de unidade de medida (centímetro em vez de metro ou vice-versa), registro de peso como altura ou vice-versa e registro da medida de outra criança ^28^. A preferência pelos dígitos 0 e 5 sugere arredondamento. Já a preferência por outro dígito indica a construção fictícia do registro dos dados ^9^. No caso do SISVAN, como a preferência ocorre para os dígitos 0 e 5, o principal indicativo é que esse resultado ocorre devido a arredondamento.

O local de execução também pode representar fonte de erros devido a espaço físico insuficiente, sem privacidade, com temperaturas extremas, pouca iluminação, piso irregular e parede com rodapé ^27^. Em estudo conduzido por Lima et al. ^29^ na APS em Alagoas, 60% dos estabelecimentos de assistência à saúde não tinham local apropriado para a realização de antropometria. Entre os locais utilizados, destacavam-se salas de curativo, de vacinação, de espera e até mesmo corredores. Na população pediátrica, a utilização de roupas pesadas, fraldas, adereços na cabeça e o comportamento pouco colaborativo da criança e de seus cuidadores representam potenciais fontes de erro ^18^^,^^27^. Considerando a aferição da altura, crianças de até 24 meses devem ser medidas deitadas e crianças com 24 meses ou mais devem ser medidas em pé. Em inquéritos internacionais, por exemplo, se uma criança de até 24 meses for medida em pé, 0,7cm deve ser acrescentado à sua altura. Se uma criança com mais de 24 meses for medida deitada, então se desconta 0,7cm de sua altura ^18^^,^^21^. No SISVAN, não há campo para preencher a informação sobre a posição de aferição da altura da criança.

A frequência de VBI para altura foi quase o dobro da frequência de VBI para peso, e a variabilidade entre os municípios também foi mais expressiva no índice da altura. Teoricamente, a variabilidade da altura seria menor que a variabilidade do peso, considerados dois aspectos: a tendência secular de crescimento da altura ^30^ e a possibilidade de aumento ou redução do peso corporal decorrente dos ciclos e eventos de vida ^31^. A descrição do procedimento para aferição das variáveis antropométricas apresentada na documentação do SISVAN ^3^^,^^7^^,^^24^ indica que a aferição do peso depende de uma balança calibrada e colocada sobre superfície nivelada, bem como das vestimentas da criança. A medida da altura, por sua vez, depende de um estadiômetro adequado instalado em local apropriado para aferir a altura de crianças. Adicionalmente, é necessário que o profissional da saúde posicione a cabeça da criança no plano de Frankfurt e observe cinco pontos anatômicos da criança para garantir a execução completa do procedimento. Em nossa análise, a maior frequência de VBI para altura ocorreu entre crianças de 12 a 23 meses, sugerindo que, nessa fase do desenvolvimento, as crianças apresentam movimentos motores mais independentes ^32^, o que pode dificultar para o profissional mantê-la na posição adequada enquanto realiza a leitura da medida no cursor.

De modo geral, observamos que a frequência de VBI e o DP foram menores entre crianças com 2 anos ou mais. Bilukha et al. ^33^ também observaram que o percentual de valores implausíveis e o DP dos índices nutricionais eram menores entre crianças de 24 a 59 meses. Desigualdades sociais e regionais já foram observadas como determinantes em outros aspectos relacionados ao estado nutricional infantil ^31^. Em nossa análise, o erro de medida antropométrica foi menos frequente nas regiões Sul e Sudeste.

Durante o período de 2008 a 2020, houve redução da frequência de VBI, porém, em 2020, a frequência para altura desse indicador ainda era superior ao limite de 1%. Um aspecto que reforça a tese de que a qualidade dos dados antropométricos é insuficiente é o fato de que havia municípios nos quais a média do índice da altura era similar à média do padrão de crescimento, mas com o DP alcançando o dobro do DP do padrão de crescimento. Nossos resultados indicam avanços na qualidade dos dados antropométricos no período analisado, mas eles ainda são insuficientes para se atingir o padrão de qualidade de mensuração recomendado pela OMS. O investimento em ações coordenadas e direcionadas para a melhoria da coleta e do registro de dados antropométricos tem se mostrado efetivo para a redução da preferência de dígito ^34^^,^^35^, da frequência de valores implausíveis ^35^ e da dispersão dos dados ^35^.

Entre as limitações deste trabalho, destacamos o número limitado de variáveis sociodemográficas das crianças, ausência de informações relativas às condições dos estabelecimentos de saúde (estrutura física e equipamentos) e às capacitações e habilidades dos profissionais da saúde na realização do exame antropométrico. A inconsistência da variável raça/cor acrescentou, ainda, limites para a análise das desigualdades na distribuição do VBI. Por exemplo, Finaret & Hutchinson ^36^ avaliaram as diferenças entre crianças com e sem dados de altura completos e biologicamente plausíveis em 116 DHS. Entre as diferenças observadas, destacamos que as crianças com dados completos de altura e biologicamente plausíveis eram mais velhas, viviam em áreas urbanas, tinham mães com maior escolaridade e maior riqueza familiar. Na Índia, Dwivedi et al. ^37^, utilizando análise multinível, identificaram que a maior contribuição para a variabilidade dos índices nutricionais estava localizada no nível comunidade. Após incluir a informação referente à equipe antropométrica como nível subordinado à comunidade, os autores observaram uma redução marginal e significativa da dispersão dos índices nutricionais.

Em nossa análise, alguns elementos devem ser pontuados. O primeiro é o número de crianças menores de 5 anos de todos os municípios brasileiros que contribuíram com informações antropométricas para esta análise: 23.453.620. O segundo foi a utilização dos indicadores recomendados pela OMS para avaliação da qualidade dos dados antropométricos propostos, à princípio, para inquéritos nutricionais e adaptados para um sistema de informação em saúde. Salientamos que alguns indicadores como razão sexo, arredondamento da idade e normalidade (assimetria e curtose) não foram apresentados, tendo em vista os limites naturais da comunicação científica. Acreditamos que essa decisão não afetou nossas conclusões sobre os resultados. O terceiro foi a proposta das faixas de adequação para o DP dos três índices nutricionais A-I, P-I e IMC-I, utilizando dados de 158 inquéritos de crianças menores de 5 anos representativos de suas respectivas populações e uma metodologia robusta para estimar as faixas de adequação. Esses três aspectos contribuíram para aumentar a consistência da análise, em nossa opinião.

Grandes bases de dados provenientes de sistemas de informação em saúde têm sido utilizadas em outros países com diferentes objetivos, tais como avaliar a tendência de obesidade em crianças de 2 a 4 anos nos Estados Unidos ^38^, produzir uma curva de crescimento na França ^39^ e avaliar a tendência e os fatores sociodemográficos associados ao excesso de peso e à obesidade na Espanha ^40^. Nesse sentido, o SISVAN apresenta potencial ainda pouco explorado tanto para utilização no planejamento de ações em âmbito territorial ^41^ quanto para produção científica.

Os valores observados dos indicadores, como preferência de dígito superior a 20, frequência de VBI superior a 1% e sobredispersão dos índices nutricionais, indicam que os dados têm qualidade insuficiente e que, possivelmente, a realização de estimativas de diagnósticos nutricionais em âmbito populacional para crianças menores de 5 anos pode ser um problema caso nenhum tipo de ajuste prévio seja conduzido.

A curto prazo, com os devidos ajustes estatísticos, será possível utilizar os dados antropométricos do SISVAN para calcular estimativas das prevalências dos diagnósticos nutricionais e, assim, analisar as tendências do estado nutricional infantil. Experiência bem-sucedida nesse sentido já foi conduzida com registros antropométricos de gestantes ^42^. A médio e longo prazo, são necessários investimentos adicionais específicos na vigilância alimentar e nutricional com garantia de financiamento adequado para ações coordenadas e direcionadas, como aquisição de equipamentos apropriados, padronização da técnica de mensuração e utilização das informações antropométricas no planejamento, no monitoramento e na avaliação das ações de alimentação e nutrição na APS.
